# Crosstalk of Oxidative Phosphorylation-Related Subtypes, Establishment of a Prognostic Signature and Immune Infiltration Characteristics in Colorectal Adenocarcinoma

**DOI:** 10.3390/cancers14184503

**Published:** 2022-09-16

**Authors:** Can Wang, Guoliang Cui, Dan Wang, Min Wang, Qi Chen, Yunshan Wang, Mengjie Lu, Xinyi Tang, Bolin Yang

**Affiliations:** 1 Department of Colorectal Surgery, Affiliated Hospital of Nanjing University of Chinese Medicine, Jiangsu Province Hospital of Chinese Medicine, Nanjing 210004, China; 2 Department of Gastroenterology, The Second Affiliated Hospital of Nanjing University of Chinese Medicine, Nanjing 210017, China

**Keywords:** colorectal adenocarcinoma, oxidative phosphorylation, prognosis predicting, tumor microenvironment

## Abstract

**Simple Summary:**

Oxidative phosphorylation (OXPHOS) plays an important role in the progression of colorectal adenocarcinoma (COAD). The aim of our study was to investigate the expression pattern of OXPHOS-related genes (ORGs), and an OXPHOS-related prognostic signature was constructed to classify COAD patients into high-risk and low-risk groups. Then, we analyzed the relationship between risk scores and tumor microenvironment, somatic mutation, and efficacy of immunotherapy and chemotherapy. Additionally, a nomogram was established by combining clinical features and risk scores, and its predictive ability was verified by receiver operating characteristics and calibration curves. Overall, the OXPHOS-related signature can be used as a reliable prognostic predictor of COAD patients.

**Abstract:**

Oxidative phosphorylation (OXPHOS) is an emerging target in cancer therapy. However, the prognostic signature of OXPHOS in colorectal adenocarcinoma (COAD) remains non-existent. We comprehensively investigated the expression pattern of OXPHOS-related genes (ORGs) in COAD from public databases. Based on four ORGs, an OXPHOS-related prognostic signature was established in which COAD patients were assigned different risk scores and classified into two different risk groups. It was observed that the low-risk group had a better prognosis but lower immune activities including immune cells and immune-related function in the tumor microenvironment. Combining with relevant clinical features, a nomogram for clinical application was also established. Receiver operating characteristic (ROC) and calibration curves were constructed to demonstrate the predictive ability of this risk signature. Moreover, a higher risk score was significantly positively correlated with higher tumor mutation burden (TMB) and generally higher gene expression of immune checkpoint, N6-methyladenosine (m6A) RNA methylation regulators and mismatch repair (MMR) related proteins. The results also indicated that the high-risk group was more sensitive to immunotherapy and certain chemotherapy drugs. In conclusion, OXPHOS-related prognostic signature can be utilized to better understand the roles of ORGs and offer new perspectives for clinical prognosis and personalized treatment.

## 1. Introduction

Colorectal cancer (CRC) is the world’s top three malignancies in terms of incidence rate and mortality, respectively, accounting for 10.0% and 9.4% of the total incidence rate and mortality of cancer that occurred in 2020 [1]. CRC is heterogeneous and involves many pathogenic mechanisms, including somatic mutation, gene fusion, genetic instability and epigenetic changes [2,3,4]. In the new CRC diagnosis, metastatic disease occurred in 20% of patients, and metastatic disease later occurred in another 25% of patients with localized disease [5]. For advanced CRC, even when resection is used in combination with modern adjuvant systemic therapy, only 20% of patients can be cured and 70% of patients relapse [6]. There are few treatment options for CRC patients with metastatic disease, and some molecular-targeted drugs are only suitable for patients with specific mutation characteristics [7]. Even responders will inevitably develop drug resistance and then relapse [8]. The evaluation of tumor molecular biomarkers is helpful to identify the histological origin of the tumor and predict the risk of tumor progression or metastasis and recurrence, but the prediction ability of traditional risk assessment is limited [9,10]. COAD is the most common and widely studied pathological type of CRC [11]. Therefore, we investigated reliable biomarkers of OXPHOS for immunotherapy, and a novel signature for prognosis prediction in patients with COAD.

Altered energy metabolism is one of the “hallmarks of cancer” [12]. The Warburg effect suggests that glycolysis is the main way of tumor cell metabolism and the enhancement of glycolysis in tumor cells is due to the irreversible damage of mitochondrial OXPHOS [13,14]. However, metabolic processes considered to be downregulated in cancer, such as OXPHOS, act as a modulator in tumor energy metabolism and are closely related to prognosis [15,16]. In addition, it has also been reported that OXPHOS is not only used by cancer cells but also upregulated in some tumors [17,18]. Inhibiting OXPHOS function by reducing hypoxia and improving anti-tumor immune response may change the tumor microenvironment [19]. Therefore, it may be a promising investigation to target OXPHOS in future cancer prevention and treatment [20,21]. It has been proved that inhibition of OXPHOS is a promising new therapy and OXPHOS-related genes (ORGs) may be prognostic factors for CRC [22,23]. Increased copy number of mitochondrial DNA (mtDNA) promotes cell proliferation, migration, and metastasis in CRC with microsatellite stable phenotypes through enhancement of mitochondrial OXPHOS [24]. Moreover, OXPHOS was shown to be associated with increased drug resistance in CRC cells [25].

With the advancement in next-generation sequencing technology, OXPHOS has been reported to be used in risk prediction models for lung adenocarcinoma and uterine corpus endometrial carcinoma [26,27]. Therefore, we aim to identify ORGs as prognostic markers of COAD and a risk score signature of ORGs was then established and validated to provide possible guidance for immunotherapy in COAD patients.

## 2. Materials and Method

### 2.1. Data Acquisition

The data of RNA sequence transcriptome data, mutation and clinical in patients with COAD were downloaded from the Cancer Genome Atlas (TCGA, https://portal.gdc.cancer.gov/, accessed on 1 December 2021), which included 514 cases in the TCGA-COAD dataset. The RNA-Seq data included 473 tumor samples and 41 normal samples, after removing the samples with incomplete prognostic information, a total of 417 COAD patients were enrolled for further analysis. Classification criteria for left-sided and right-sided COAD [28]: The tumor primary sites in the cecum, ascending colon, and hepatic flexure are right-sided COAD. The tumor primary sites in splenic flexure, descending colon, sigmoid colon, and rectosigmoid junction are left-sided COAD.

### 2.2. Identification of OXPHOS-Related Prognostic Signature

A total of 289 OXPHOS-related genes (ORGs) were obtained by searching published studies [26,27]. Differentially expressed genes (DEGs) in the TCGA-COAD dataset were screened using the “limma” package [29]. The cutoff values were |log fold change (logFC)| > 1 and *p* < 0.05 and the results were summarized in a Venn plot and heatmap.

### 2.3. Functional and Gene Set Enrichment Analysis

Gene Ontology (GO) function and Kyoto Encyclopedia for Genes and Genomes (KEGG) pathway enrichment analysis were performed to reveal the functions of DEGs using the package “clusterProfiler” [30]. In addition, based on the MSigDB database, gene set enrichment analysis (GSEA) was also carried out to identify the biological processes between the two risk groups.

### 2.4. Construction of Risk Score Signature

COAD patients were randomly divided into three sets including a training set (*n* = 209), testing set (*n* = 208) and entire set (*n* = 417) at the ratio of 1:1. The clinical detail of tumor patients is shown in Table S1 and there is no significant difference among these three groups. Univariate Cox regression analysis was performed on the training set to identify DEGs related to prognosis [31]. Using multivariate Cox regression analysis and Lasso analysis, the risk score of each sample was calculated by using the expression value of the key genes and weighting its corresponding coefficient. The risk score calculation formula is as follows: risk score = $\sum \mathrm{coef}*\mathrm{Exp}\left(\mathrm{genes}\right),$ (coef: coefficient of gene; Exp (genes): expression of genes). According to the risk score calculation formula, COAD patients with different risk scores were selected into two risk groups (high or low) using the median risk score as the threshold. Subsequently, Kaplan–Meier survival analysis was used to reveal the prognostic differences between the two risk groups and the independent prognostic value of the risk score [32]. The clinical outcomes of COAD patients were predicted by constructing a nomogram using the “rms” package. ROC curve analysis was conducted to evaluate the predictive ability of risk score and other clinical factors. Calibration curves were generated to evaluate the consistency between the predicted and actual survival rates.

### 2.5. Cell Culture

Three human colorectal cancer cell lines (Caco-2, HT-29, HCT-116) were all purchased from the China Center for Type Culture Collection (CCTCC, Wuhan, China). The normal colon epithelial cell line (FHC) was obtained from the Cell Bank of Type Culture Collection of the Chinese Academy of Sciences (Shanghai, China). HT-29, HCT-116, FHC, Caco-2 were cultured in McCoy’s 5A, RPMI-1640, and DMEM, respectively, (Gibco, Shanghai, China) which was supplemented with 10% fetal bovine serum (FBS, Gibco, Shanghai, China) and 1% antibiotics. All cells were incubated at 37 °C with 5% CO_2_.

### 2.6. Quantitative RT-PCR

TRIZOL reagent (Thermo, Waltham, MA, USA) was used to extract total RNA, and complementary DNA (cDNA) was synthesized with Revert Aid First Strand cDNA Synthesis kit (Vazyme, Nanjing, China) according to the manufacturer’s instructions. Quantitative RT-PCR was performed using ChamQ Universal SYBR qPCR Master Mix (Vazyme, Nanjing, China) and the relative expression of the target gene was analyzed using the 2^−ΔΔCT^ method. β-actin was chosen as the internal reference. The primer sequences are listed in Table S2.

### 2.7. Tumor Microenvironment (TME) Analysis

TME is mainly composed of tumor cells, immune cells, stromal cells and extracellular matrix [33], and the difference could be explored by using the ESTIMATE algorithm [34]. Package “gsva” was applied to quantify the infiltrating score of immune cells and immune-related function by single-sample gene set enrichment analysis (ssGSEA) [35]. Multiple databases (TIMER, CIBERSORT, CIBERSORT-ABS, QUANTISEQ, MCPCOUNTER, XCELL, and EPIC) were used to assess the difference between tumor-infiltrating immune cells (TIICs) and the components of 21 TIICs were evaluated between two risk groups by the CIBERSORT algorithm [36]. The correlation between risk score, ORGs expression and TIICs was demonstrated by Pearson analysis.

### 2.8. Mutational Analysis

Somatic mutation and microsatellite instability (MSI) information were obtained from the TCGA database. The relationship between TMB, MSI and risk score, and the differences between TMB and MSI in two risk groups were revealed [37]. The top 20 genes mutation were exhibited in a waterfall map using the package “maftools” [38]. As previously mentioned, one class logistic regression (OCLR) was performed to calculate the stem cell indices of each COAD sample [39].

### 2.9. Immunophenoscore (IPS) and Chemotherapy Analysis

Immunophenotypic scores were calculated based on the expression value of representative genes, such as CTLA4, PD-L1, and the results of IPS were obtained from the Cancer Immunome Atlas (TCIA) [40]. The half-maximal inhibitory concentration (IC50) of representative drugs was assessed through a database named Genomics of Drug Sensitivity in Cancer (GDSC) [41]. The correlation between ORGs expression and drug sensitivity was estimated through the NCI-60 database [42].

### 2.10. Statistical Analysis

Continuous variables were described as mean with standard error (SD); categorical variables were represented as frequencies. The differences between the two risk groups were calculated by a Student’s *t*-test or Chi-squared test. Kaplan–Meier analyses were used to calculate the difference in overall survival (OS). The relationship analysis was calculated by the Pearson correlation test. *p* < 0.05 was considered statistically significant.

## 3. Results

### 3.1. Differentially Expressed Oxidative Phosphorylation-Related Genes

**Figure 1:**
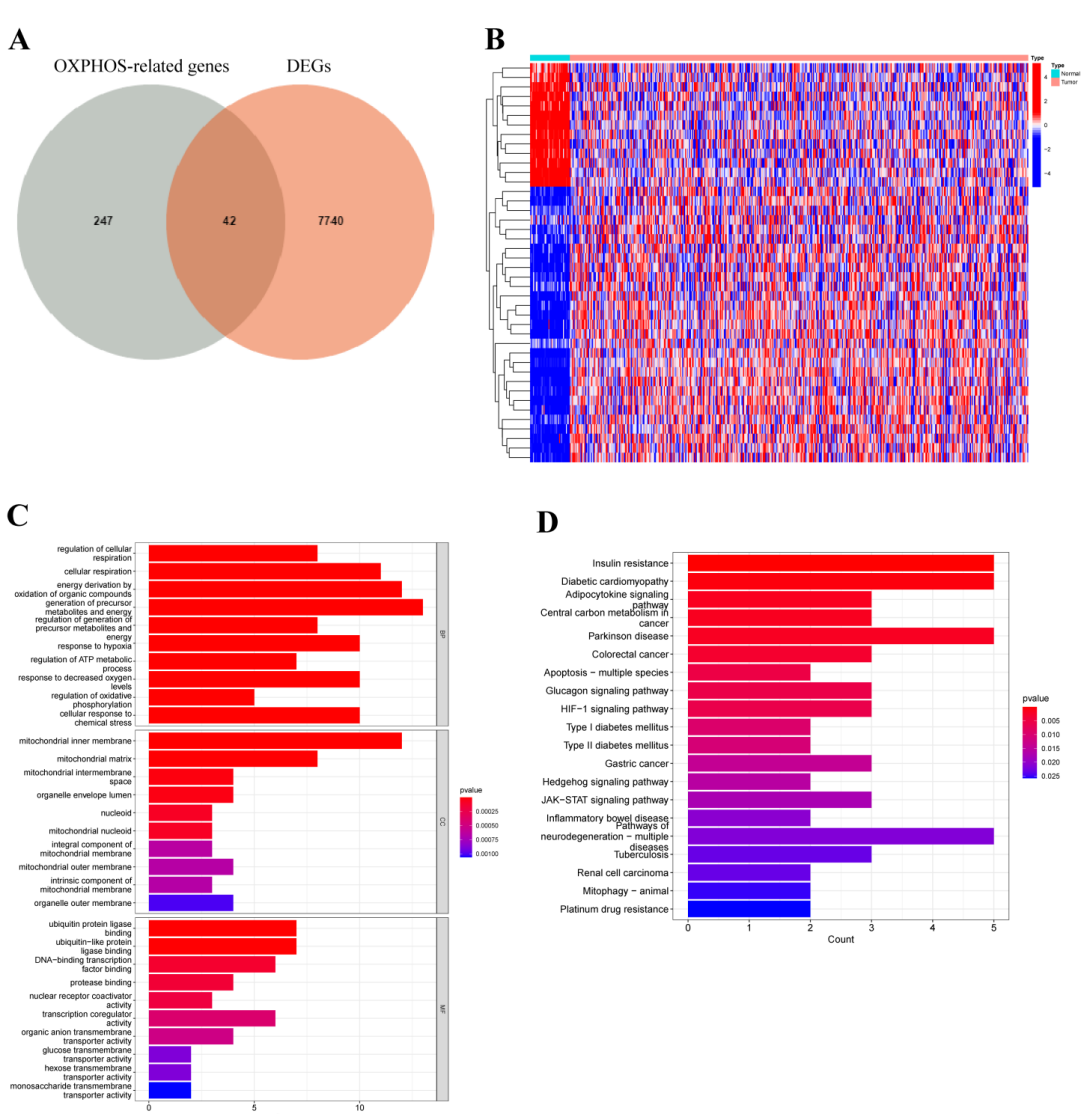
Differential expression analysis and functional annotation of oxidative phosphorylation (OXPHOS)-related gene in patients with COAD. (**A**) Venn diagram revealed the distribution of DEGs and ORGs in COAD. (**B**) The heatmap depicted the different expressions of ORGs between tumor and normal tissues. Blue blocks indicated lower expression and red blocks represented higher expression. (**C**,**D**) GO terms and KEGG pathway enriched in differentially expressed ORGs.

Considering the complex relationship between OXPHOS and the development of COAD [43], the differences in OXPHOS-related genes (ORGs) between tumor and normal samples deserve our investigation. Therefore, we performed differential expression analysis of 289 ORGs to explore the role of OXPHOS in COAD. Firstly, after the intersection of 7782 differentially expressed genes (DEGs) from TCGA, the Venn plot and heatmap identified 42 differentially expressed ORGs, of which 13 genes were downregulated and 29 genes were upregulated (Figure 1A,B). Next, the biological function of DEGs was explored. The GO term enrichment analysis showed that DEGs were correlated with the regulation of cellular respiration, cellular respiration and energy derivation by oxidation of organic compounds in biological process (BP) group, mitochondrial inner membrane, mitochondrial matrix and mitochondrial intermembrane space in cellular component (CC) group, ubiquitin protein ligase binding, ubiquitin-like protein ligase binding and DNA-binding transcription factor binding in molecular function (MF) group (Figure 1C). Additionally, KEGG pathways results manifested that DEGs are primarily associated with adipocytokine signaling pathway, central carbon metabolism in cancer, and colorectal cancer (Figure 1D).

### 3.2. Prediction Signature Construction

**Figure 2:**
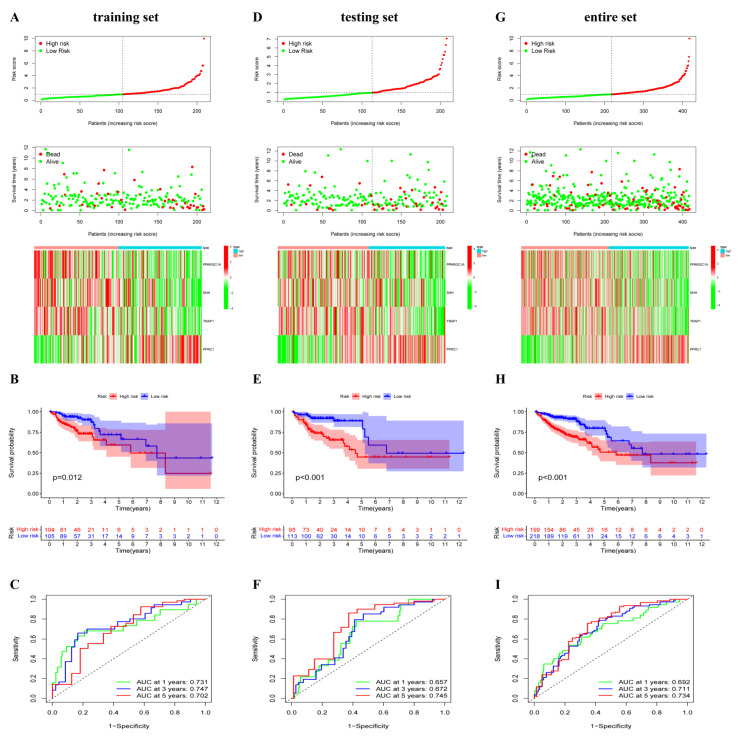
Construction and validation of the oxidative phosphorylation (OXPHOS)-related gene prognostic signature in the training, testing and entire set. (**A**,**D**,**G**) The risk curve displayed risk score distribution of high-risk and low-risk groups. Scatter plot distributed the survival status and survival time, heatmap showed the expression profiles of four ORGs in high-risk and low-risk groups. (**B**,**E**,**H**) Kapan-Meier survival curves for overall survival (OS) of patients in high-risk and low-risk groups. (**C**,**F**,**I**) ROC analysis of risk score in predicting prognoses.

To investigate the prognostic role of 42 differentially expressed ORGs, 417 COAD patients were randomly divided into the training set (*n* = 209), testing set (*n* = 208) and entire set (*n* = 417). In the training set, univariate Cox regression analysis found that eight ORGs genes were identified to be substantially correlated with overall survival (OS) (Table 1). FSCN1 and PPRC1 were the dangerous elements risk factors (hazard ratio, HR > 1, *p* < 0.05). Meanwhile, MRPS23, PPARGC1A, SHH, TRAP1, MPC1 and PPA1 were the protective elements (HR < 1, *p* < 0.05, Table 1). To construct a novel OXPHOS-related prognostic signature, LASSO and multivariate Cox regression analyses were performed (Figure S1). Consequently, PPARGC1A, SHH, TRAP1 and PPRC1 were included in the signature and the risk score of COAD patients was calculated as follows: risk score = (−0.5348 × PPARGC1A expression) + (−0.4661 × SHH expression) + (−1.4386 × TRAP1 expression) + (1.1488 × PPRC1 expression). In the training set, patients with COAD were ranked from low risk to high risk and stratified into two different risk groups based on the median risk score. Furthermore, the survival time and survival status of COAD patients were shown in scatter plots, and the expression levels of four key ORGs in two risk groups were shown in a heatmap (Figure 2A). Compared to patients with higher risk scores, those with lower risk scores had significantly better survival outcomes by Kaplan–Meier analysis (*p* = 0.012, Figure 2B). The area under the curve (AUC) for 1-year, 3-year, and 5-year OS were 0.731, 0.747 and 0.702, respectively (Figure 2C), indicating that the OXPHOS-related prognostic signature had a high prediction sensitivity. Furthermore, to validate the expression level of four ORGs, qRT-PCR was used to investigate the expression of four ORGs in CRC cells (Caco-2, HT-29 and HCT-116) in in vitro experiments. Compared with FHC cells, the expression of PPARGC1A was significantly lower in Caco-2 cells, while upregulated in HCT-116 cells. SHH expression was dramatically upregulated in HT-29 cells, but there was no such trend in Caco-2 or HCT-116 cells. TRAP1 expression was significantly higher in Caco-2 cells and HT-29 cells, and PPRC1 expression was significantly upregulated in three CRC cells (Figure S2A–D).

### 3.3. OXPHOS-Related Prognostic Signature Validation

To validate the predictive ability of this signature, we performed the same analysis in testing and entire sets. In both sets, COAD patients with different risk groups were identified based on the same calculation formula. Survival information and the expression level of four key ORGs can be effectively distinguished according to this signature in COAD patients (Figure 2D,G). Similarly, patients in the high-risk group presented worse OS than those in the low-risk group using the Kaplan–Meier analysis (Figure 2E,H). The AUCs at 1-, 3- and 5-years were 0.657, 0.672 and 0.745 in the testing dataset, respectively; 0.692, 0.711 and 0.734 in the entire dataset (Figure 2F,I). These results demonstrated good predictive performance of the OXPHOS-related prognostic signature in patients with COAD.

### 3.4. Prediction Value of OXPHOS-Related Prognostic Signature

**Figure 3:**
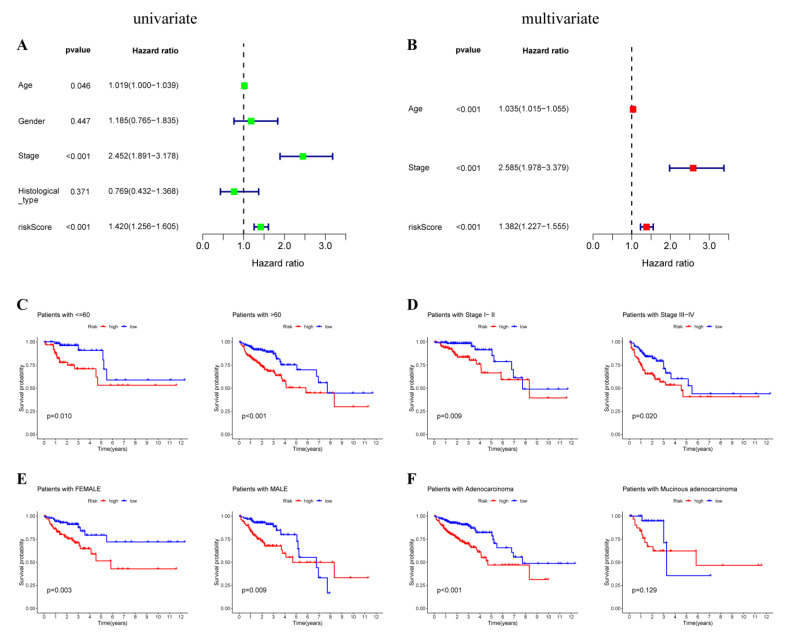
Subgroup analysis of the prognostic value of risk score in COAD patients. Identification of independent factors by univariate (**A**) and multivariate (**B**) Cox regression analysis. Prognostic value of risk score in patients with different age (**C**), different stage (**D**), different gender (**E**) and different histological_types (**F**).

To investigate whether risk score could be used as an independent prognostic factor, univariate and multivariate Cox regression analyses were performed. The results indicated that risk score was an independent prognostic indicator, and a higher level of risk score was related to a worse prognosis for COAD patients (Figure 3A,B). Besides, subgroup survival analysis was performed to evaluate the predictive value of the signature in clinical conditions. Similarly, Kaplan–Meier analysis reveals that a lower risk score was correlated with better OS in male or female patients, younger or older patients, stage I and II or stage III and IV and patients with adenocarcinoma (Figure 3C–F). However, perhaps because of the small sample size, there was no significant difference in survival rate between high-risk and low-risk groups in patients with mucinous colorectal adenocarcinoma (Figure 3F). In addition, survival analysis revealed that a higher risk score correlated with worse prognoses in left-sided and right-sided COAD patients (Figure S3). The risk score was higher in right-sided than that in left-sided COAD patients (Figure S4), suggesting a worse prognosis in the right-sided COAD patients, which was consistent with previous reports [44]. In aggregate, these results indicated that the risk model is a promising prognostic classifier for COAD patients.

**Figure 4:**
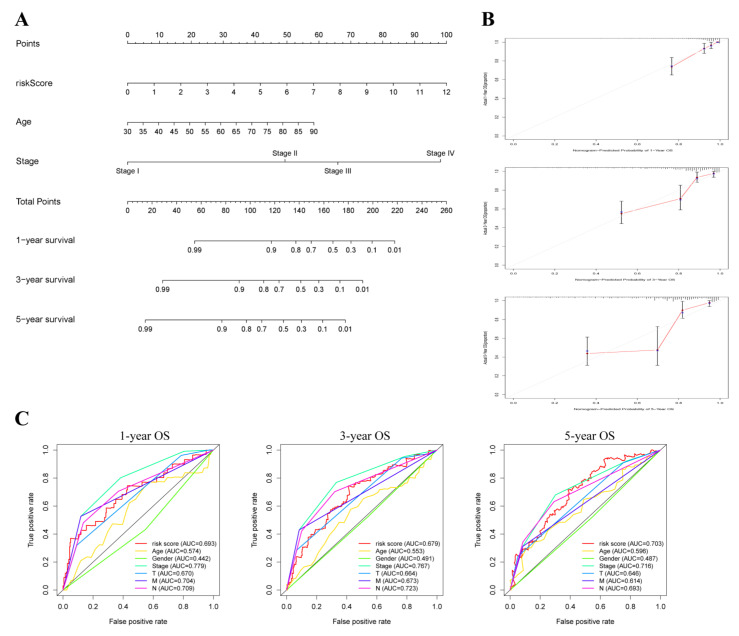
Construction of nomogram for predicting survival of COAD patients. (**A**) The prediction of 1-, 3-, and 5-year survival for COAD patients based on the prognostic nomogram including clinic-pathological features (age, stage, risk score). (**B**) Calibration plots of 1-, 3-, and 5-year OS revealed the consistency between nomogram-predicted survival probabilities and the actual outcome. (**C**) ROC curves of 1-, 3-, and 5-year OS indicated the prognostic accuracy of OXPHOS-related gene risk score and other clinical characteristics (age, gender, clinical stage).

To accurately predict the OS of COAD patients, we constructed a nomogram of 1-, 3- and 5-year survival probabilities based on the risk score and clinicopathological characteristics (Figure 4A). The consistency of actual and predicted OS in COAD patients was revealed using the calibration curve (Figure 4B). According to the ROC curves, the OXPHOS-related risk score had better AUC values compared to other clinical factors (Figure 4C).

### 3.5. GESA, ESTIMATE and ssGSEA Analysis

**Figure 5:**
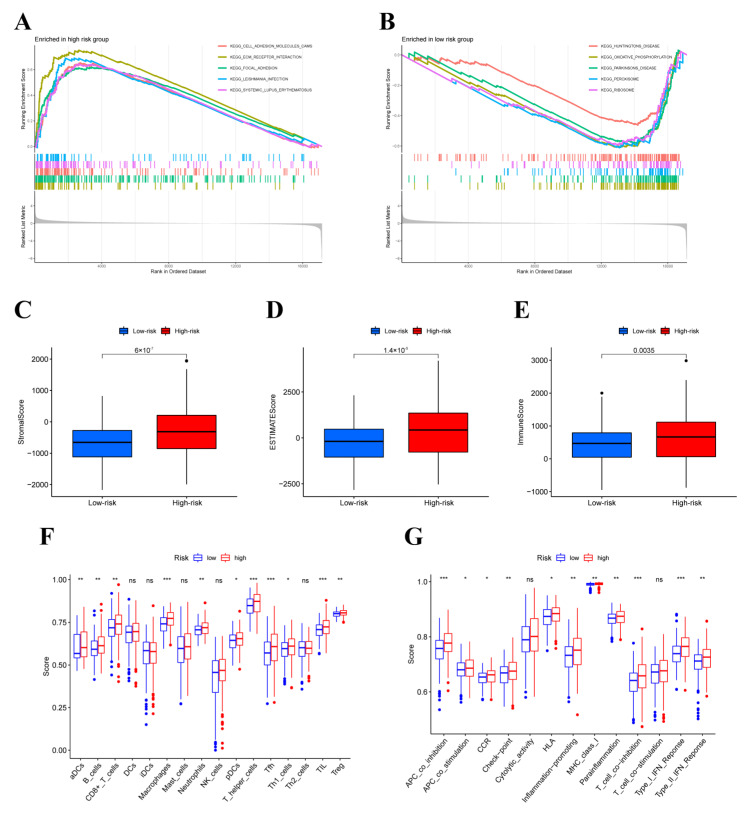
The gene set enrichment analysis, and estimation of immune cell infiltration in different risk groups. GSEA was performed to predict the potential functions and pathways in high-risk group (**A**) and low-risk group (**B**). (**C**–**E**) stromal score, immune score and ESTIMATE scores in high-risk and low-risk groups were shown. (**F**,**G**) The difference between immune cells and immune-related functions between two risk groups. Adjusted *p*-values were shown as ns, not significant, * *p* < 0.05, ** *p* < 0.01, *** *p* < 0.001.

To elucidate the potential tumor-related pathway between two risk groups, the GSEA analysis was performed. The results showed that in the high-risk group, adhesion molecules CAMs, ECM receptor interaction, and focal adhesion were mainly enriched, while oxidative phosphorylation, peroxisome, and ribosome were primarily enriched in the low-risk group (Figure 5A,B). Additionally, we performed ESTIMATION and ssGSEA analyses to identify the potential relationship between immune status and risk scores. The ESTIMATION analysis revealed that the score of stromal, immune, and ESTIMATE in the low-risk group was lower, which indicated that the high-risk group had lower tumor purity (Figure 5C–E). The results of ssGSEA confirmed that the high-risk group had higher levels of immune cell infiltration and more active immune-related functions (Figure 5F,G).

### 3.6. Tumor-Infiltrating Immune Cells (TIICs) Analysis

**Figure 6:**
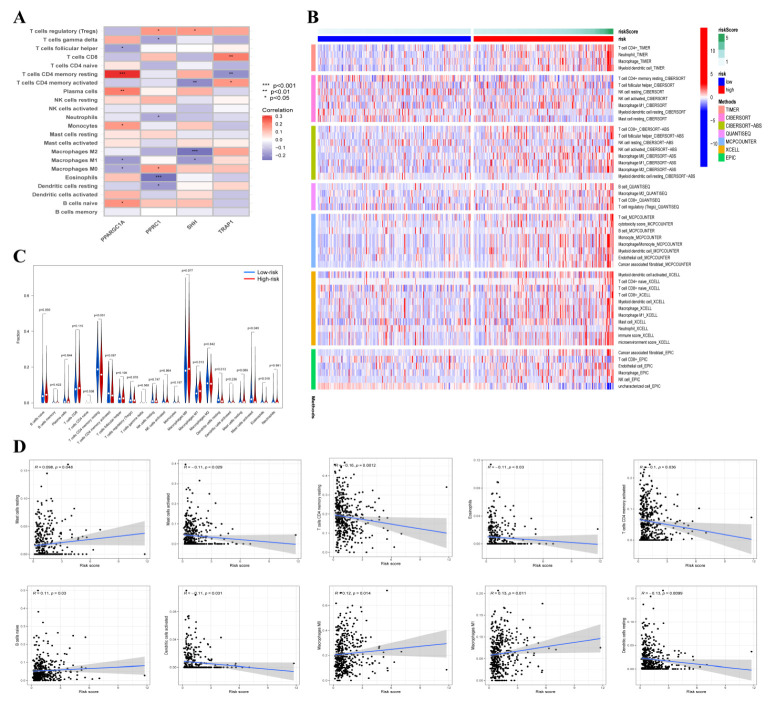
The correlation between risk score and tumor-infiltrating immune cell (TICs). (**A**) The relationship between four OXPHOS-related gene expression level and TICs. Red indicates positive correlation while blue indicates negative correlation. (**B**) The infiltration of 21 types of immune cells in high-risk and low-risk groups was estimated by TIMER, CIBERSORT, CIBERSORT-ABS, QUANTISEQ, MCPCOUNTER, XCELL and EPIC database. (**C**) Comparison of TICs in high-risk and low-risk groups. (**D**) The correlation between risk score and immune cells, and the results significant differences were shown.

We further explore the function of the tumor microenvironment in COAD patients with different risk scores. The correlation between four ORGs expressions and TIICs was explored by using the TIMER database (Figure 6A). Furthermore, the immune infiltration level of 22 types of immune cells was compared between two groups using multiple databases (Figure 6B). In the low-risk group, the fractions of resting CD4 memory T cells, resting dendritic cells and activated mast cells were significantly higher. Additionally, naïve B cells and M1 macrophages were distributed more in the high-risk group (Figure 6C). The relationship between risk score and TIICs was further evaluated and risk score was positively correlated with four TIICs but was negatively correlated with six TIICs (Figure 6D). The above results demonstrated that the risk scores in this signature discriminate the different features of TIICs in patients with COAD.

### 3.7. Somatic Variation Analysis

**Figure 7:**
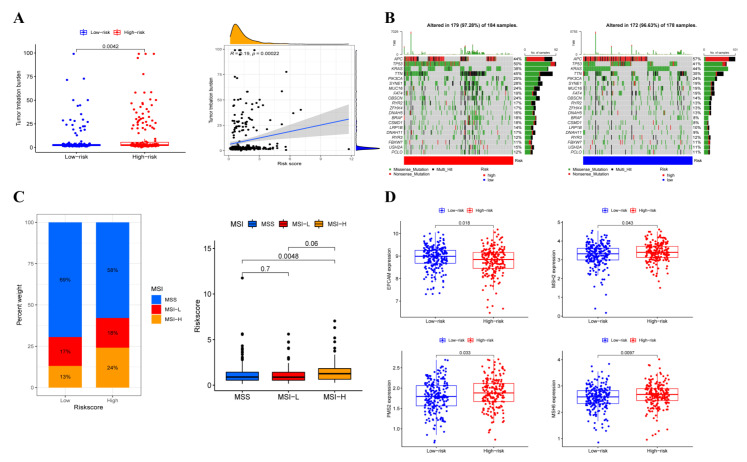
The relationship between TMB and risk score. (**A**) The difference between TMB in high-risk and low-risk groups and the correlation between TMB and risk score. (**B**) Waterfall plot revealed the mutation information of genes with high mutation frequency in high-risk and low-risk groups. (**C**) The proportion of different microsatellite states and the risk score of three microsatellite states in different risk groups. (**D**) The expression of EPCAM, MSH2, PMS2 and MSH6 in two risk groups.

Survival outcomes of cancer immunotherapy are associated with tumor mutation load (TMB). We investigated the correlation between risk score and TMB level; as shown in Figure 7A, the TMB level in the low-risk group was significantly lower and was significantly positively related to risk scores. Next, differences in the distribution of somatic variants were also analyzed. A waterfall plot was used to exhibit the top 20 highest mutated genes (Figure 7B). As a tumor immune marker, microsatellite instability (MSI) can be used to evaluate the effect of immunotherapy. A higher portion of patients belonged to the MSI-high (MSI-H) subtype and a lower proportion of patients belonged to the microsatellite stability (MSS) in the high-risk group. In addition, patients had significantly higher risk scores in the MSI-H subgroup than those in the MSS subgroup (Figure 7C). Moreover, the expression level mismatch repair (MMR) genes were also detected and the results revealed that EPCAM expression was upregulated while MSH2, PMS2 and MSH6 expression were downregulated in the low-risk group (Figure 7D).

### 3.8. Immunotherapy Efficacy Analysis

**Figure 8:**
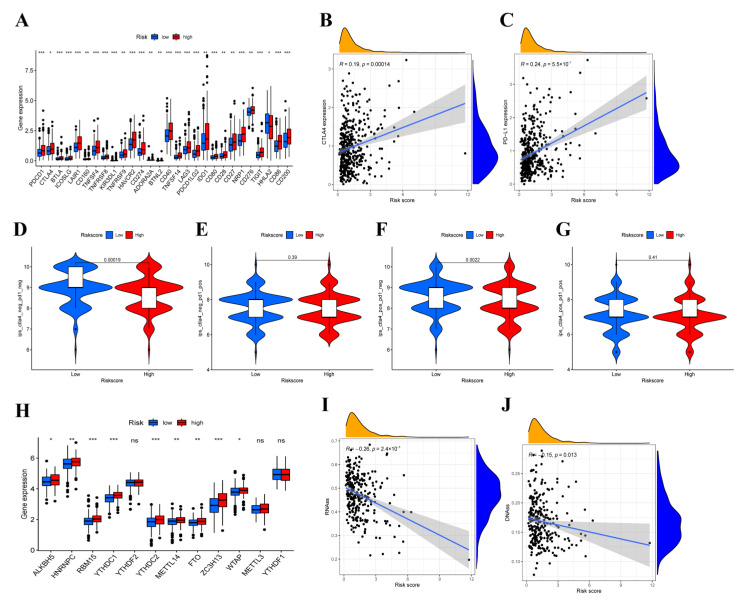
The results of immune checkpoint and immunophenoscore (IPS)**.** (**A**)The expression of immune checkpoint genes and the correlation between risk score and CTLA4 expression (**B**) and PD-L1 expression (**C**). (**D**–**G**) The differences of IPS in patients with different risk score. (**H**) The expression level of main genes of m6A in high-risk and low-risk groups. (**I**,**J**) Relationship between risk score and RNAss, DNAss. Adjusted *p*-values were shown as ns, not significant, * *p* < 0.05, ** *p* < 0.01, *** *p* < 0.001.

To reveal the role of the risk score in predicting the immunotherapy efficacy of COAD patients, the expression level of immune checkpoint genes was analyzed. We found significant differences in 28 of 47 immune checkpoint genes expression, most of which were generally significantly higher in the high-risk group (Figure 8A). Moreover, the risk score showed a positive correlation with CTLA4 and PD-L1 expression (Figure 8B,C). The results mentioned above indicated that patients with higher risk scores promised to respond to immunotherapy, and immunosuppressants acted on immune checkpoints, such as CTLA4, and PD-L1, and may be applied for immunotherapy in COAD patients. The quantitative scoring scheme of immunophenoscores (IPS) can be used to determine the determinants of immunogenicity in tumors and can serve as an effective predictor for detecting the responses of anti-PD-1 and anti-CTLA4-antibodies [40]. Therefore, to evaluate the possibility of receiving immune checkpoint inhibitors (ICIs) treatment, we calculated the IPS scores and as shown in Figure 8D–G, the score of IPS-CTLA4-neg-PD1-neg and IPS-CTLA4 in the low-risk group was significantly higher than that in the high-risk group, while no statistically significant difference was observed on IPS-CTLA4-neg-PD1-pos and IPS-CTLA4-pos-PD1-pos scores. In addition, differential expression analysis was conducted on m6A regulators to identify the key mediators using TCGA-COAD datasets. Compared with the low-risk group, it was found that the expression of ALKBH5, HNRNPC, RBM15, YTHDC1, YTHDC2, METTL14, FTO, ZC3H13 and WTAP were significantly higher in the high-risk group (Figure 8H). Moreover, a significant negative correlation was revealed between the two stemness scores and risk scores (Figure 8I,J). The results demonstrated the efficiency of the OXPHOS-related prognostic signature in the prediction of immunotherapy and helped to clarify the mechanism underlying the different prognoses of patients with different risk scores.

### 3.9. Chemotherapy Sensitivity Analysis

**Figure 9:**
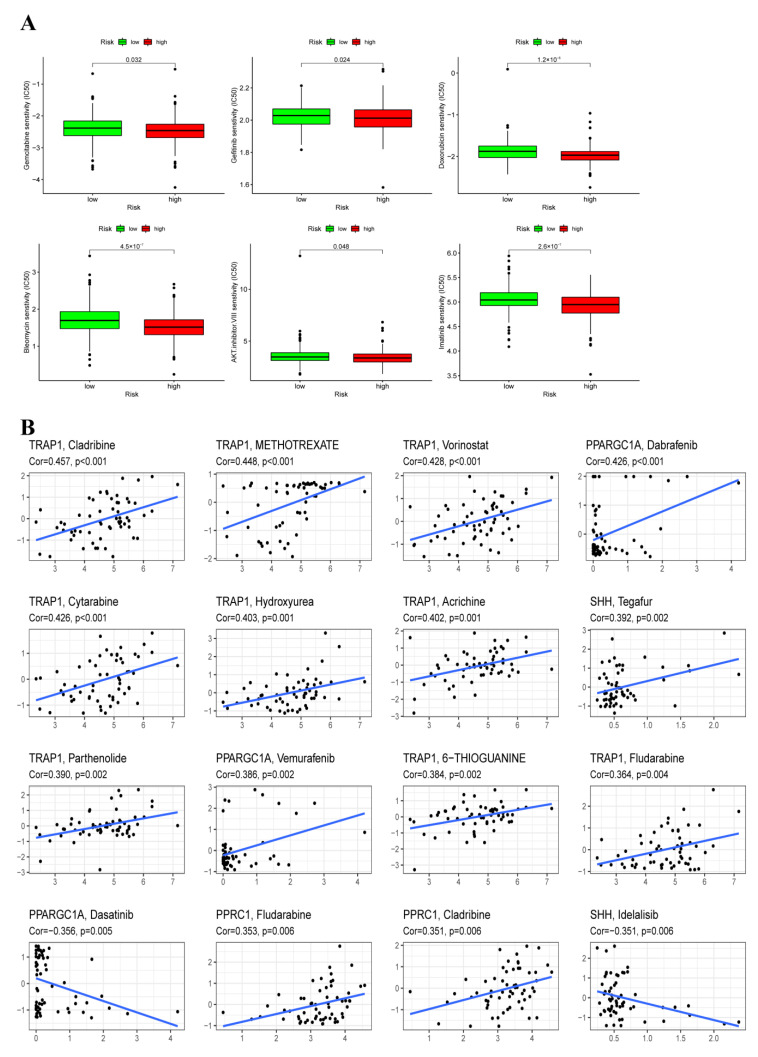
Prediction of the response to common chemotherapy drugs between the low-risk and high-risk groups. (**A**) Estimation of IC50 value for Gemcitabine, Gefitinib, Cisplatin, Bleomycin, AKT. inhibitor. VIII, Imatinib. (**B**) Scatter plot of the association between three ORGs expression and drug sensitivity.

To further explore the difference in chemotherapeutic drugs resistance potential, we compared the estimated IC50 level of chemotherapy drugs including Gemcitabine, Gefitinib, Cisplatin, Bleomycin, and AKT.inhibitor.VIII, Imatinib. As for the high-risk group, the sensitivity to these representative drugs was better than that of the low-risk group (Figure 9A). In addition, a significant association was also found between the four ORGs expression and the sensitivity of some chemotherapeutic drugs (Figure 9B). For instance, SHH expression level was positively associated with increased resistance to tegafur but negatively correlated with increased susceptibility to idelalisib.

## 4. Discussion

Non-metastatic COAD patients are mostly treated with surgery, while patients with advanced recurrent or metastatic colorectal cancer are often treated with re-surgery, chemotherapy, radiotherapy, targeted therapy, or other comprehensive treatment interventions [5]. Currently, despite advances in the diagnosis and treatment of CRC, the prognosis of patients with CRC remains alarming [45]. In terms of drug therapy, 5-fluorouracil (5-FU) is currently the classic agent for palliative and adjuvant systemic chemotherapy for colorectal cancer, but the benefits that patients receive from 5-FU-based therapies are often compromised by the development of chemotherapeutic resistance [46,47]. In this study, an OXPHOS-related prognostic signature was established to predict the prognosis, quantify the tumor immune environment and guide the treatment strategies for immunotherapy for COAD patients.

We obtained the clinical information and ORGs expression files from the TCGA-COAD database. As a result, four key DEGs (PPARGC1A, SHH, TRAP1, PPRC1) were finally identified as prognosis predictor genes, these genes were included in the calculation formula to establish OXPHOS-related prognostic signature and verified by an ROC curve. The risk score based on four ORGs could independently predict the prognosis. Moreover, the OXPHOS-related nomogram provided a predicted survival possibility of 1/3/5-year OS.

Tumor necrosis factor receptor-associated protein 1 (TRAP1), a paralog of the HSP90 molecular chaperone is well known to be involved in mitochondrial respiration regulation. Previous studies demonstrated that TRAP1 may be an anti-tumor molecular target and a key regulator of the reprogramming of energy metabolism in tumor cells, indicating that the change of mitochondrial bioenergy mediated by TRAP1 plays a more common role in tumorigenesis [48]. Elevated TRAP1 levels were associated with malignant progression and metastasis in COAD [49]. Moreover, multiple studies have confirmed that overexpression of TRAP1 can protect cancer cells from various antitumor drugs [50,51]. Peroxisome proliferator-activated receptor gamma, coactivator 1 alpha (PPARGC1A) was reported to promote metastasis through mediating mitochondrial biogenesis and oxidative phosphorylation in cancer cells [52]. It is well known that mitochondrial DNA (mtDNA) is involved in the production of adenosine triphosphate (ATP) via OXPHOS. The number and time of tumor formation were related to the degree of mtDNA consumption, and long-term depletion leads to the damage of mtDNA replication thus inducing the expression of early development and survival-promoting markers, including sonic hedgehog (SHH) [53]. PPARG related coactivator 1 (PPRC1), a transcription factor and regulator of mitochondrial biogenesis, may become a new therapeutic target for glioblastoma [54,55]. The above studies clarify the functions of these four genes in oxidative phosphorylation and the roles in tumors. Our study promoted the relationship between ORGs and the prognosis of COAD.

Previous studies have shown that compared with left-sided CRC, right-sided CRC presents lower morbidity, a larger tumor size, lower grade of histological differentiation, deeper invasion and worse prognosis [56]. Right-sided CRC was associated with more frequency of microsatellite instability (MSI) and CpG island hypermethylation phenotype (CIMP), higher mutational load and a more complex mutation spectrum, such as KRAS, PIK3CA, BRAF, PTEN and GNAS mutations [57]. While left-side CRC presents more chromosomal instability (CIN) and aneuploidy, and mutations in APC are more common [56]. In this study, right-sided COAD patients had a higher risk score than left-side COAD patients, and a higher risk score was related to worse OS, which was consistent with the worse prognosis of right-side COAD. In addition, TMB level, gene mutation frequency and MSI-high proportion were higher in patients with a higher risk score, which may explain the difference in mutation profiles and MSI between left-sided and right-sided COAD. These results indicated that the risk score has a good discrimination ability between right-sided and left COAD.

Consensus Molecular Subtype (CMS) classification divided CRC into four subtypes [58]. CMS1 tumors present hypermutation and MSI-H. Moreover, the common infiltrating immune cells were mainly Th1 cells, cytotoxic T cells and NK cells, and immunodetectable molecules, such as CTLA4, PD1 and PD-L1 were highly expressed with high immunogenicity in CMS1 tumors [59]. In this study, the results of ssGSEA confirmed that the high-risk group had higher levels of immune cell infiltration (such as Th1 cells), which were consistent with features of CMS1 tumors. Moreover, TMB level, CTLA4 and PD-L1 expression were significantly positively correlated with risk scores and COAD patients had significantly higher risk scores in the MSI-H subgroup than those in the MSS subgroup. It could be speculated that the high-risk group in OXPHOS-related signatures may serve as a predictive model tool for CRC patients with CMS1. Furthermore, the most striking feature of CMS3 tumors is the altered metabolic profile and reprogramming of cellular metabolism [59]. OXPHOS is a regulator of tumor energy metabolism and is involved in glycolysis in tumor cells [60]. The OXPHOS-related signature may also be suitable for the assessment of the tumor immune microenvironment and prognostic prediction in CMS3 tumors.

The relationship between the tumor microenvironment and OXPHOS-related signatures was confirmed using the ESTIMATE algorithm, which showed that the proportion of resting CD4 memory T cells, resting dendritic cells and activated mast cells was increased, while the proportion of naïve B cells and M1 macrophages was reduced in the low-risk group. Macrophages are innate immune cells, and metabolic reprogramming is the main factor affecting the different phenotypes of macrophages [61]. M1 macrophages rely mainly on glycolysis rather than on the tricarboxylic acid (TCA) cycle and OXPHOS activation [62]. In this study, risk scores were positively correlated with the fraction of M1 macrophages, speculating that patients may be more prone to OXPHOS injury with the increase of the risk score.

It was reported that metabolic reprogramming accelerated the proliferation, invasion, metastasis and chemoresistance of tumor cells, even promoting immune escape in COAD [63,64,65]. Many therapeutic strategies targeting the Warburg effect have been reported, but all of them are faced with the problems of high cytotoxicity or low sensitivity due to the loss of certain metabolic enzymes after treatment [66,67]. Microsatellite stable COAD is characterized by immune immunity type and immune desert type, as well as low tumor lymphocyte infiltration level and tumor mutational burden (TMB), which is also considered a typical “cold tumor” and poor effect on immunotherapy [68,69]. This study showed that patients with lower risk scores were more likely to have cold tumor subtypes, higher IPS analysis scores, and worse immunotherapy effects. At the same time, the analysis of immune checkpoints also proves that the patients with lower risk scores have worse immunotherapy effects. Similarly, worse sensitivity to certain representative drugs including Gemcitabine, Gefitinib, Bleomycin, and AKT.inhibitor.VIII, Imatinib occurred in patients with lower risk scores.

Nevertheless, there are still some shortcomings in our study. The data were not validated by an external clinical cohort. Secondly, the conclusion of this study may be biased due to incomplete records of some clinical indicators. Furthermore, the biological functions of these prognosis-related ORGs should be further determined by cell function assays in COAD.

## 5. Conclusions

In a nutshell, this study constructs a prognostic signature associated with OXPHOS, which may help predict survival and assess the possible benefits of treatment, thus improving the OS of COAD patients. These results may also help to identify new immune biomarkers or targeted therapies, as well as new insights into the occurrence and progression of COAD.

## Figures and Tables

**Table 1:** Univariate COX regression analysis of eight OXPHOS-related genes in COAD in the training set.

Genes	HR	Low 95%CI	High 95%CI	*p*-Value
MRPS23	0.499	0.255	0.976	0.042
PPARGC1A	0.546	0.307	0.972	0.040
SHH	0.595	0.365	0.972	0.038
FSCN1	1.346	1.052	1.721	0.018
TRAP1	0.437	0.205	0.931	0.032
MPC1	0.433	0.214	0.878	0.020
PPRC1	2.288	1.098	4.766	0.027
PPA1	0.516	0.266	1.000	0.050

## Data Availability

The datasets presented in this study can be found in online repositories. The names of the repositories and accession numbers can be found in the article or Supplementary Materials.

## Supplementary Materials

The following supporting information can be downloaded at: https://www.mdpi.com/article/10.3390/cancers14184503/s1, Figure S1: Prognostic value of ORGs in TCGA-COAD training set. (A,B) Multivariate Cox regression via LASSO is presented, and three candidate ERGs were selected in training cohort. Figure S2: Expression validation of four ORGs. (A-D) qRT-PCR analysis of the mRNA expression levels of four ORGs in different cell lines. Adjusted *p*-values were shown as ns, not significant, * *p* < 0.05, ** *p* < 0.01, *** *p* < 0.001. Figure S3: Prognostic value of risk score in patients with left-sided and right-sided COAD. Figure S4: The difference in risk score between left-side and right-sided COAD patients. Table S1: Clinical features of COAD patients. Table S2: Primer sequence of genes in qRT-PCR.
