# Supplementary material for: Crosstalk of Oxidative Phosphorylation-Related Subtypes, Establishment of a Prognostic Signature and Immune Infiltration Characteristics in Colorectal Adenocarcinoma
Source: Cancers (Basel). 2022 Sep 16;14(18):4503. doi: 10.3390/cancers14184503 (PMC9496738; doi:10.3390/cancers14184503)
Supplement: Supplementary file 1 [file cancers-14-04503-s001.zip › cancers-1912847-supplementary/supplementary table.pdf]

Table S1 Clinical features of COAD patients

Covariates	Type	Total	Test	Train	Pvalue
Age	<=65	175(41.97%)	93(44.71%)	82(39.23%)	0.301
Age	>65	242(58.03%)	115(55.29%)	127(60.77%)	
Gender	FEMALE	193(46.28%)	91(43.75%)	102(48.8%)	0.349
Gender	MALE	224(53.72%)	117(56.25%)	107(51.2%)	
Stage	Stage I	72(17.27%)	37(17.79%)	35(16.75%)	0.900
Stage	Stage II	160(38.37%)	79(37.98%)	81(38.76%)	
Stage	Stage III	117(28.06%)	60(28.85%)	57(27.27%)	
Stage	Stage IV	57(13.67%)	26(12.5%)	31(14.83%)	
Stage	unknow	11(2.64%)	6(2.88%)	5(2.39%)	
T	T1	9(2.16%)	2(0.96%)	7(3.35%)	0.349
T	T2	74(17.75%)	40(19.23%)	34(16.27%)	
T	T3	284(68.11%)	141(67.79%)	143(68.42%)	
T	T4	49(11.75%)	24(11.54%)	25(11.96%)	
T	unknow	1(0.24%)	1(0.48%)	0(0%)	
M	M0	311(74.58%)	155(74.52%)	156(74.64%)	0.658
M	M1	57(13.67%)	26(12.5%)	31(14.83%)	
M	unknow	49(11.75%)	27(12.98%)	22(10.53%)	
N	N0	247(59.23%)	124(59.62%)	123(58.85%)	0.896
N	N1	98(23.5%)	47(22.6%)	51(24.4%)	
N	N2	72(17.27%)	37(17.79%)	35(16.75%)	

Table S2 Primer sequence of genes in qRT-PCR

	Forward sequence	Reverse sequence
β-actin	CATGTACGTTGCTATCCAGGC	CTCCTTAATGTCACGCACGAT
PPARGC1A	TCTGAGTCTGTATGGAGTGACAT	CCAAGTCGTTCACATCTAGTTCA
SHH	CTCGCTGCTGGTATGCTCG	ATCGCTCGGAGTTTCTGGAGA
TRAP1	AGGACGACTGTTCAGCACG	CCGGGCAACAATGTCCAAAAG
PPRC1	CAAGCGCCGTATGGGACTTT	GGAGGCATCCATGTAGCTCT
