# Supplementary figures and images for: Crosstalk of Oxidative Phosphorylation-Related Subtypes, Establishment of a Prognostic Signature and Immune Infiltration Characteristics in Colorectal Adenocarcinoma
Source: Cancers (Basel). 2022 Sep 16;14(18):4503. doi: 10.3390/cancers14184503 (PMC9496738; doi:10.3390/cancers14184503)

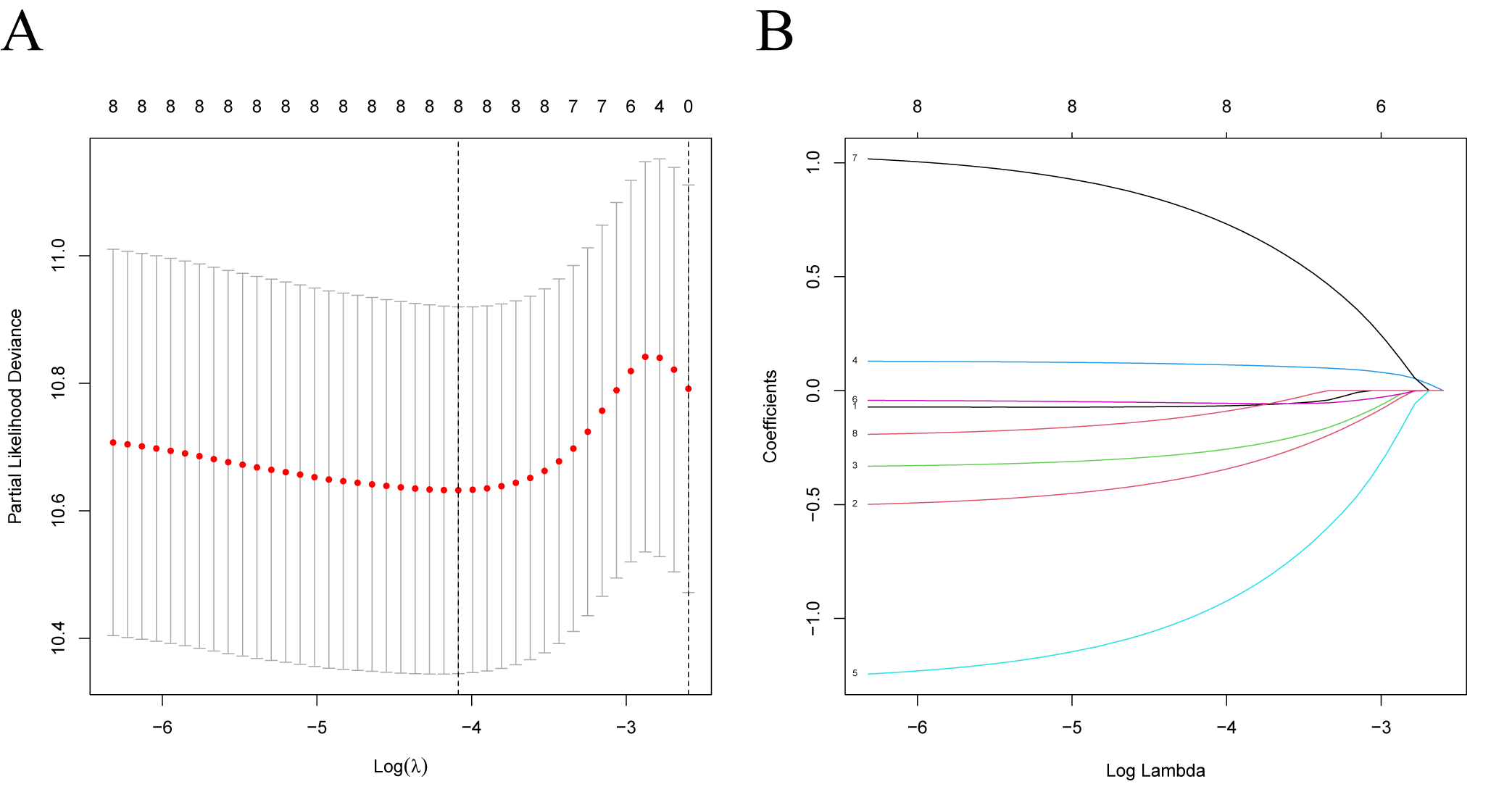

Supplement: Supplementary file 1 [file cancers-14-04503-s001.zip › cancers-1912847-supplementary/FIGS1.tif]

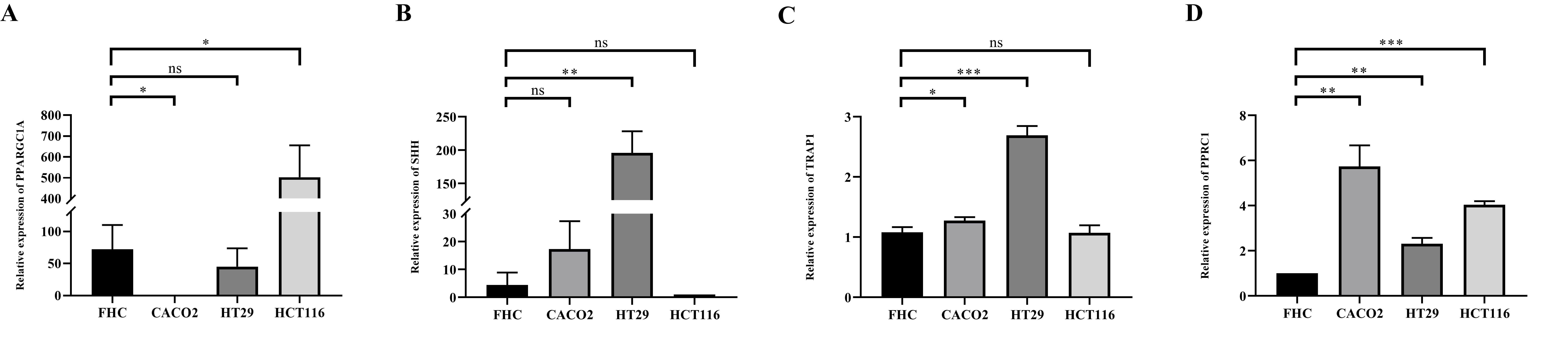

Supplement: Supplementary file 1 [file cancers-14-04503-s001.zip › cancers-1912847-supplementary/FIGS2.tif]

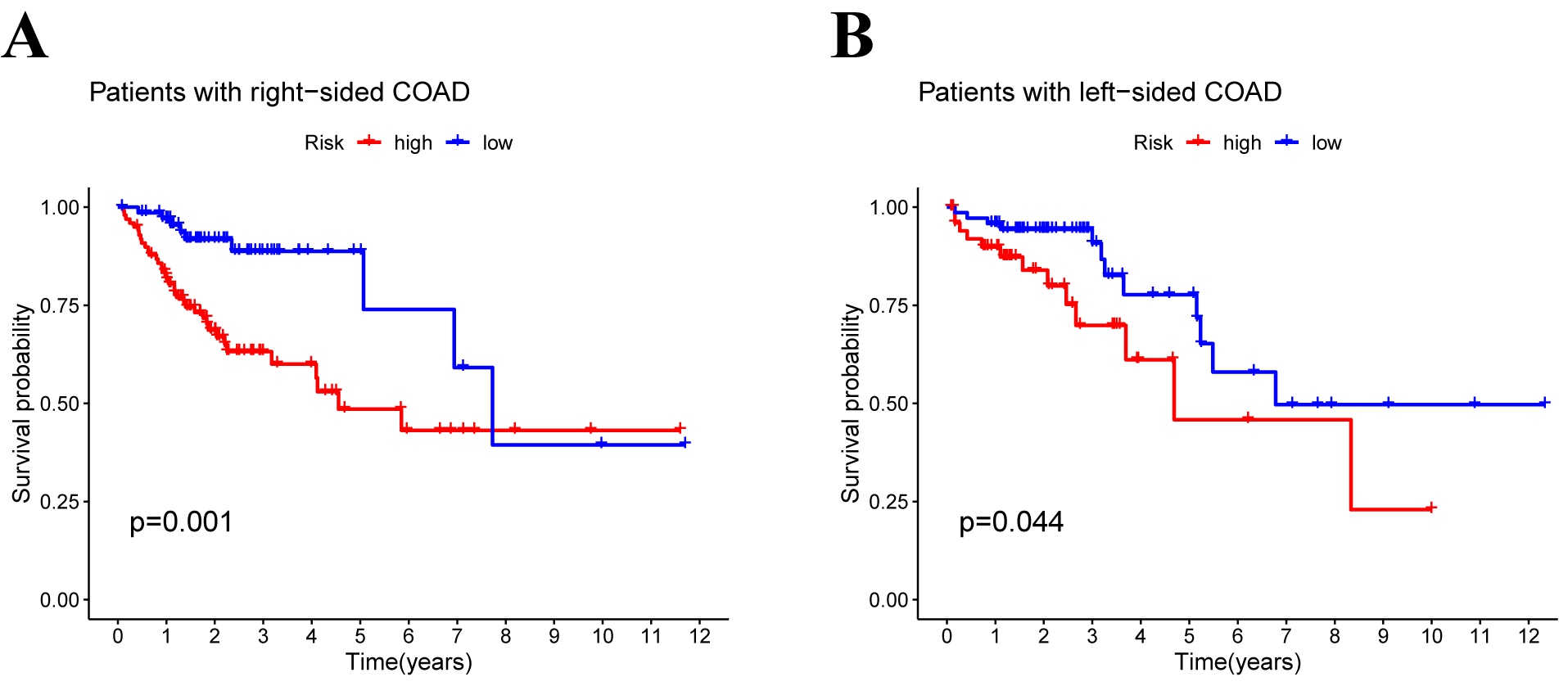

Supplement: Supplementary file 1 [file cancers-14-04503-s001.zip › cancers-1912847-supplementary/FIGS3.tif]

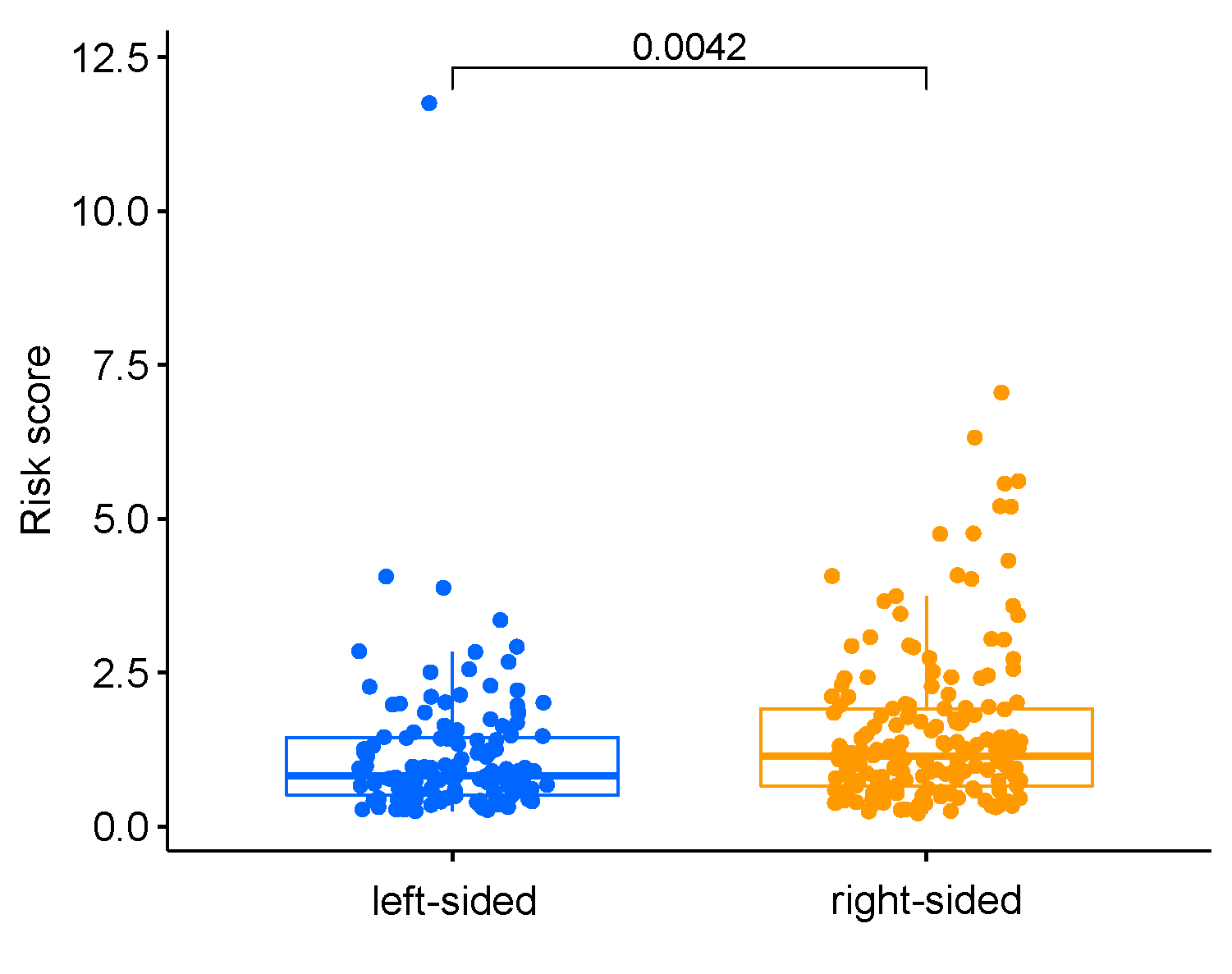

Supplement: Supplementary file 1 [file cancers-14-04503-s001.zip › cancers-1912847-supplementary/FIGS4.tif]
